# Differentiation of Benign and Malignant Thyroid Nodules with ANFIS by Using Genetic Algorithm and Proposing a Novel CAD-Based Risk Stratification System of Thyroid Nodules

**DOI:** 10.3390/diagnostics13040740

**Published:** 2023-02-15

**Authors:** Ahmet Cankat Ozturk, Hilal Haznedar, Bulent Haznedar, Seyfettin Ilgan, Osman Erogul, Adem Kalinli

**Affiliations:** 1 Institute of Natural Science, Department of Biomedical Engineering, TOBB University of Economics and Technology, 06560 Ankara, Türkiye; 2 Institute of Natural Science, Department of Computer Engineering, Erciyes University, 38280 Kayseri, Türkiye; 3 Department of Computer Engineering, Gaziantep University, 27310 Gaziantep, Türkiye; 4 Department of Nuclear Medicine, Ankara Guven Hospital, 06540 Ankara, Türkiye; 5 Presidency Office, Rectorate, Middle East Technical University, 06800 Ankara, Türkiye

**Keywords:** thyroid, thyroid nodule, classification, ANFIS, deep neural network, guideline

## Abstract

The thyroid nodule risk stratification guidelines used in the literature are based on certain well-known sonographic features of nodules and are still subjective since the application of these characteristics strictly depends on the reading physician. These guidelines classify nodules according to the sub-features of limited sonographic signs. This study aims to overcome these limitations by examining the relationships of a wide range of ultrasound (US) signs in the differential diagnosis of nodules by using artificial intelligence methods. An innovative method based on training Adaptive-Network Based Fuzzy Inference Systems (ANFIS) by using Genetic Algorithm (GA) is used to differentiate malignant from benign thyroid nodules. The comparison of the results from the proposed method to the results from the commonly used derivative-based algorithms and Deep Neural Network (DNN) methods yielded that the proposed method is more successful in differentiating malignant from benign thyroid nodules. Furthermore, a novel computer aided diagnosis (CAD) based risk stratification system for the thyroid nodule’s US classification that is not present in the literature is proposed.

## 1. Introduction

Nodules are defined as discrete lesions in the thyroid tissue that are distinguished radiologically from the normal thyroid parenchyma. The incidence rates of thyroid nodules in the adult population are reported as 10–67% depending on the technique used [1]. On the other hand, the probability of malignancy of thyroid nodules is reported to be between 1.6% and 12% in different series [2].

Ultrasonography (US) is considered to be the most widely-used and most effective imaging technique in the evaluation of thyroid nodules [3,4,5,6,7,8,9,10,11,12,13,14,15]. Imaging methods such as Magnetic Resonance (MR), Positron Emission Tomography (PET), Scintigraphy, and Computed Tomography (CT) are of limited importance in the evaluation of thyroid nodules.

The gold standard method in the differential diagnosis of thyroid nodules is fine-needle aspiration biopsy (FNAB). Although FNAB is considered an accurate and cost-effective method with significant diagnostic sensitivity and specificity [16,17,18], the application of FNAB to all nodules creates a burden on the healthcare system and causes anxiety for most patients.

It is a current practice to divide the nodules into risk categories according to their US apparencies and offer FNAB more selectively. The current guidelines suggest different nodule size criteria for FNAB to different risk categories, and they more or less use the same criteria. American College of Radiology Thyroid Imaging Reporting and Data System (ACR TIRADS), European Thyroid Imaging, Reporting and Data System (EU TIRADS), and The Korean Thyroid Imaging Reporting and Data System (K-TIRADS) are the most widely used guidelines in the literature [2,3,17,18,19,20]. Briefly, there are three sonographic features most commonly associated with malignant nodules, which are border irregularity, a taller-than-wide pattern, and microcalcifications. Solid and hypoechoic nodules carrying one of these are considered high-risk nodules for malignancy. However, there are also some other sonographic features that might be important for risk stratification. In this study, the relationships of a wide range of US signs have been examined by using artificial intelligence methods. All of such efforts aim to decrease the number of unnecessary FNAB without compromising the correct diagnosis of malignant nodules [21].

Although US is considered the most important imaging technique in the evaluation of thyroid nodules, it is strictly dependent upon the performing physician. Although the sonographic features used in the differential diagnosis of nodules are well-known theoretically, the application of these criteria still depends on the experience of the examining physician. Therefore, the need for an objective and effective method is still increasingly critical in the US evaluation of thyroid nodules. The differential diagnosis of thyroid nodules is one of the research subjects of machine learning and deep learning studies since it is a common health problem.

This article’s contribution to science is given below:

1. The thyroid risk stratification guidelines used in the literature are prepared based on physician opinions. These guidelines classify nodules according to the sub-features of limited sonographic signs. This study aims to better the risk classification of thyroid nodules by examining the relationships of a wide range of ultrasound (US) signs by using artificial intelligence methods.
2. A novel computer aided diagnosis (CAD)-based risk stratification system for the thyroid nodule’s US classification that is not present in the literature is proposed.
3. An innovative method based on training Adaptive-Network Based Fuzzy Inference Systems (ANFIS) by using Genetic Algorithm (GA) is used to differentiate malignant from benign thyroid nodules. The comparison of the results from the proposed method to the results from the commonly used derivative-based algorithms and Deep Neural Network (DNN) methods yielded that the proposed method is found to be more successful in differentiating malignant from benign thyroid nodules.

The rest of the study is presented as follows. The general structure of materials and methods are given in Section 2. The results are demonstrated in Section 3 and Section 4 presents the conclusion.

## 2. Materials and Methods

All thyroid nodules used in this study consist of histopathologically verified benign and malignant ones. Sonographic images of thyroid nodules were retrospectively evaluated by a specialist sonographer with more than 25 years of experience in the field and the sonographic features of benign and malignant nodules were determined as original. Classification studies are carried out by using an innovative approach based on training of the ANFIS model with the GA algorithm in the differential diagnosis of malignant/benign nodules with the signs determined by the sonographer, which is considered as a real-world problem. In addition, the performance of the used innovative approach is compared to the performances of other artificial intelligence methods based on ANFIS trained with derivative-based back propagation algorithm and Deep Neural Network (DNN). In addition, the Decision Tree Algorithm is applied to determine the most effective signs in the differential diagnosis of malignant/benign nodules. In this study, a new guideline showing significant effect of the sonographic sign on the differential diagnosis of malignant/benign nodules is introduced to the literature. Valuable discussions on the performances of different methods to solve this real-world problem are presented in this paper.

### 2.1. Dataset Description

A data set covering histopathologically proven 398 thyroid nodules from 224 patients with thyroid cancer is used in this study. Total of 398 nodules were examined in this study, consisting of 284 malignant and 114 benign nodules. Patients were operated on at Güven Hospital between September 2012 and September 2016 with the cytological diagnosis of thyroid cancer. All patients have undergone a final preoperative US examination by same sonographist of multidisciplinary team, and US images were recorded prospectively. US images of patients in study group were reviewed by the same sonographist, and predefined US characteristics of all nodules, including benign ones, were recorded according to final histopathology results. This retrospective study was approved by Güven Hospital Science Committee and waived the requirement for informed consent.

Sonographic examinations, collection, and re-evaluation of all images were performed by the same sonographer with Siemens Acuson brand ultrasound system and a 12 MHz transducer. The sonographic signs of the nodules were classified under 27 categories listed in Table 1.

For P1, there is no consensus in the literature regarding its relationship with malignancy. Examination of P1 itself and its relationship with malignancy when evaluated together with other features are among the areas of interest of this study.

For P2, features that are “completely solid” and “solid-containing microcysts (<5%)” have a high risk of malignancy.

For P3, features that are “markedly hypoechoic” and “hypoechoic” have a high risk of malignancy.

For P4, if “anteroposterior/transverse diameter” is increased, it is likely to be malignant.

For P5, features that are “irregular (coarse lobulation)”, “irregular (microlobulation)”, “irregular (spiculated)” and “ill-defined” have a high risk of malignancy.

For P6, feature that is “punctate” has a high risk of malignancy.

For P7 and P8, there is no consensus in the literature regarding their relationship with malignancy. Examination of P7 and P8 themselves and their relationship with malignancy when evaluated together with other features are among the areas of interest of this study.

For P9, feature that is “hypoechoic” has a high risk of malignancy.

For P10, P11, P12, and P13, there is no consensus in the literature regarding their relationship with malignancy. Examination of P10, P11, P12, and P13 themselves and their relationship with malignancy, when evaluated together with other features, are among the areas of interest of this study.

For P14, if “interruption in echogenic capsule if there is a capsule relationship” is present or gross extrathyroidal, it is likely to be malignant.

For P15, P16, P17, and P18, there is no consensus in the literature regarding their relationship with malignancy. Examination of P15, P16, P17, and P18 themselves and their relationship with malignancy, when evaluated together with other features, are among the areas of interest of this study.

For P19, features that are “microechogenicity in the solid component” and “eccentric solid component and microechogenicity” have a high risk of malignancy.

For P20, P21, P22, P23, and P24, there is no consensus in the literature regarding their relationship with malignancy. Examination of P20, P21, P22, P23, and P24 themselves and their relationship with malignancy, when evaluated together with other features, are among the areas of interest of this study.

For P25, features that are “suspicious (<5 mm)”, “suspicious (5–10 mm)”, “suspicious (>10 mm)”, “typical metastatic (<5 mm)”, “typical metastatic (5–10 mm)” and “typical metastatic (>10 mm)” have a high risk of malignancy.

For P26, there is no consensus in the literature regarding its relationship with malignancy. Examination of P26 itself and its relationship with malignancy, when evaluated together with other features, are among the areas of interest of this study.

For P27, features that are “suspicious” and “typical” have a high risk of malignancy.

The mean age of 224 patients aged from 16 to 77 years old was 40.98, and 49 (21.875%) of the patients were male and 175 (78.125%) were female.

### 2.2. Adaptive Neuro-Fuzzy Inference System (ANFIS)

ANFIS is an artificial system developed considering Takagi Sugeno fuzzy model [22]. The created model has both the learning ability of neural networks and the ability to infer features of fuzzy logic. It computes the output by distributing the input data that it makes fuzzy with membership functions on the network with fuzzy rules. Its success in predicting problems is fairly high since this process provides inference capability to the ANFIS model. Thus, a hybrid artificial intelligence model combining two methods is developed.

**Figure 1:**
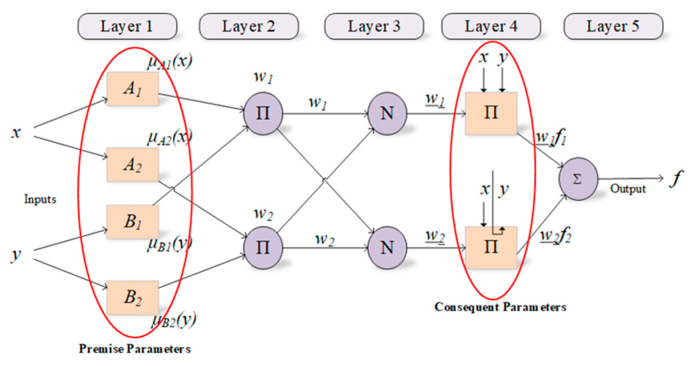
Structure of ANFIS and parameters used in training process.

ANFIS has two parts called premise and consequent. These parts are connected to each other by the fuzzy rules within the network structure of ANFIS. The main purpose of ANFIS is to optimize the parameters, called premise and consequent, that are found in these parts by using a learning algorithm with input-output datasets. In this context, parameter optimization is performed to minimize the error value between the actual and the predicted outputs during the training process [23]. In fundamental, the ANFIS structure consists of five layers described below are also depicted in Figure 1.

The fact that fuzzy systems facilitate the learning and adaptation process and that neural networks are successful in nonlinear problems by distributing control parameters on the network gives a great advantage to ANFIS architecture, which can be considered a neuro-fuzzy network model.

Training of the ANFIS model aims to produce the optimal values for the weight values depending on the input and output values. Derivative-based algorithms are commonly involved in the training of ANFIS’s parameters. However, there are difficulties such as slope calculation, as well as problems such as being unable to overcome the local minimum in derivative-based algorithms. For this reason, training ANFIS with derivative-based algorithms and updating parameters is one of the main problems. Researchers have recently proposed different algorithms not based on derivative to train the parameters of ANFIS; specifically, some of these algorithms are heuristic algorithms such as Genetic Algorithm (GA), Particle Swarm Optimization Algorithm (PSO), and Differential Development Algorithm (DE) [24]. In this study, population-based heuristic GA algorithm is implemented for training ANFIS network.

### 2.3. Genetic Algorithm

GA, whose basic principles were put forward by John Holland in the 1970s, is successfully applied to solve many types of problems [25]. GA is a heuristic algorithm used to find exact or approximate results in optimization or search problem. This algorithm was developed owing inspiration to techniques in evolutionary biology such as inheritance, mutation, selection, and crossover. GA can be applied very easily even to multidimensional problems with a large search space and number of variables. It has the ability to produce optimal results in the search spaces of problems within a reasonable period of time as a result of the tendency to try values that may be better compared to searching the whole space.

In GA, the candidate solutions that make up the population correspond to the chromosomes. These chromosomes transform into solution candidates that represent better results through various evolutionary processes. This process is continued until it reaches an acceptable fitness value, a predefined processing time, or a maximum number of generations.

**Figure 2:**
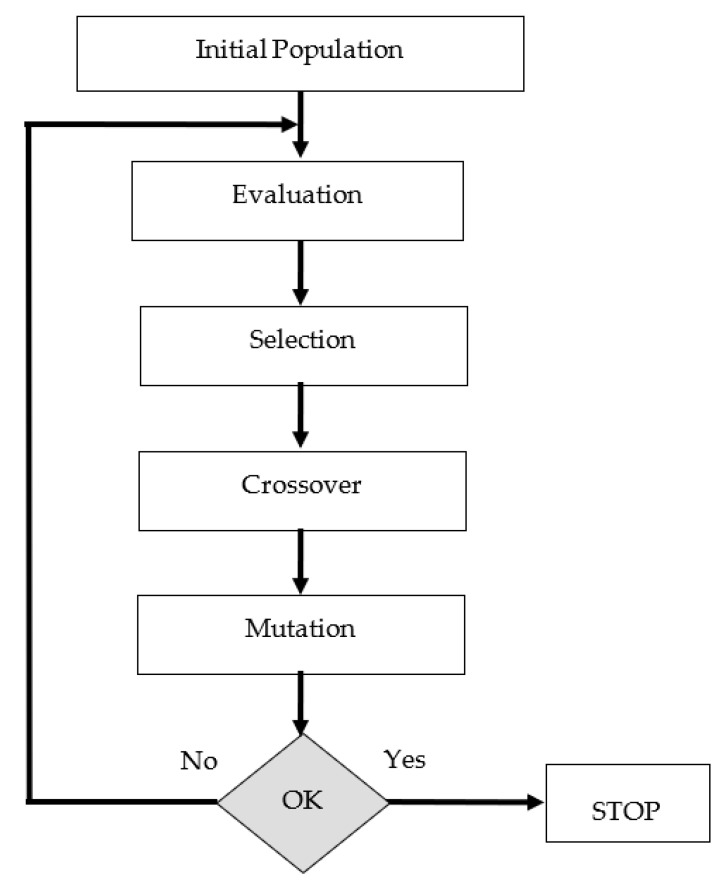
The flow chart of GA.

Chromosomes (solution candidates) are decisions that retain variables in the form of discrete or continuous values of the solution involved in GA. The fitness function is the objective function that measures the quality of chromosomes. The flowchart of GA algorithm is shown in Figure 2.

### 2.4. Training ANFIS Using the GA Algorithm

**Figure 3:**
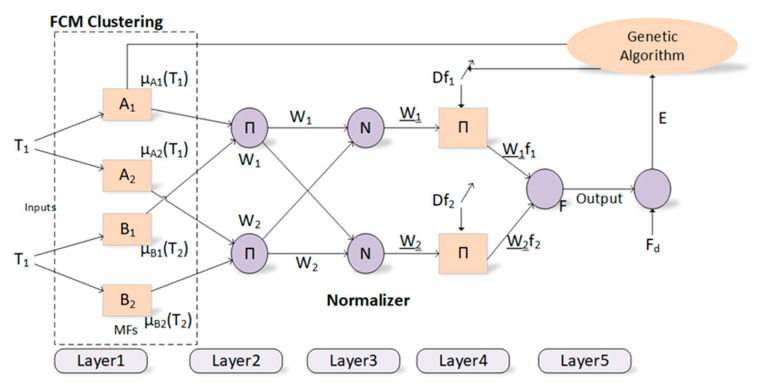
Block diagram for the proposed method.

The proposed innovative approach for training the ANFIS model with the GA algorithm is explained in detail throughout this section. Optimization of premise and consequent parameters in ANFIS has been one of the main problems, mostly due to reasons such as the slow convergence of the derivative-based algorithms, their inability to exceed the local minimum, and their dependence on the initial values to a large extent. A population-based genetic algorithm, which is a powerful algorithm that will eliminate the above-mentioned disadvantages of derivative-based algorithms, has been used to optimize the parameters of a difficult model such as ANFIS in this context. The premise and consequent parameters of ANFIS, whose initial values are generated by the FCM clustering method, represent a chromosome in the genetic algorithm. The GA tries to find the best premise and consequent parameters with the chromosome with the most appropriate fitness value considering the training process of ANFIS. The block diagram of the proposed method is depicted in Figure 3. The root mean square error (RMSE) function is used to calculate the fitness value of the solution.

During the optimization of premise and consequent parameters, the aim is to minimize the RMSE function. The most effective RMSE value is the value obtained when the actual value and the estimated value are closest to each other. The GA tries to find the most effective RMSE value until the stopping criterion is met. The number of iterations is utilized as the stopping criterion in the present study.

Classification studies have been carried out by training the ANFIS model with the GA algorithm in order to diagnose the nodules as malignant/benign (see Figure 3). The number of parameters to be optimized is related to the number of inputs, the number of membership functions, the type of membership functions and the number of rules in the training processes of the ANFIS model with the GA algorithm. Classification studies have been conducted for three different cases in this study. Three different ANFIS models used the 27, 13, and 8 sonographic signs as input. In addition, gaussmf has been used as a membership function, and 10 membership functions have been utilized for each entry within the scope of this study. Therefore, the total numbers of parameters to be optimized for three ANFIS models are 820, 400, and 250, respectively.

Training and test datasets of 398 thyroid nodules containing 27 sonographic signs are created with a random sampling method by determining the nodules as original. In this context, they are divided into two different groups as 70–30% and 80–20%. In addition, the K-fold cross validation method, one of the commonly used cross validation methods, is used to accurately evaluate the ability of the proposed method to be generalized where the objective is to repeat an experiment under independent conditions and to test the validity of its results in the K-fold cross validation method. Specifically, 5-fold and 10-fold cross-validation methods are used for data splitting in this study.

The performance of optimization algorithms largely depends on control parameters. Determination of these parameter values may vary according to the problem they are applied to, and there is no specific rule or method. Many attempts are required to determine the most appropriate control parameter values. In this context, many test attempts are performed to determine the control parameters of the GA algorithm. After these attempts, control parameters are determined as follows: the number of iterations as 100, the number of populations as 50, the crossover rate as 0.4, and the mutation rate as 0.15.

The ANFIS network is also trained with derivative-based Back Propogation (BP) and Hybrid (HB) algorithms to evaluate the performance and contribution of the proposed method over derivative-based algorithms. In this context, the learning rate for the BP algorithm and the momentum coefficient are also chosen as 0.2 and 0.4, respectively. The HB is considered as a combined method that consists of using least squares estimation and the BP algorithm. The number of iterations for the BP and HB is set as 100. Moreover, recently popular and highly successful Deep Neural Network (DNN) is utilized to diagnose the nodules as malignant/benign for comparison purpose and emphasizing the performance of the proposed method. For the simulation studies, Keras deep learning library is used to create a feed-forward neural network. Similar to the GA control parameter determination, many attempts are made to decide on the various control parameters of the DNN. Afterward, four hidden layers with 48, 36, 12, and 6 neurons per layer, respectively, are used, and the sigmoid logistic regression function is applied to the output layer. Also, the RMSprop algorithm is chosen for the optimization of the DNN model. The control parameters used in the DNN model are set as follows: the momentum coefficient as 0.9, the learning rate as 0.03, the weight decay as 0.00005, and the dropout rate as 0.2 to avoid the network from overfitting.

Commonly used Accuracy (AC), sensitivity (SN), and specificity (SP) measurements are used to evaluate the performance of the proposed method. In order to determine these measurements given in Equations (1)–(3), the expressions of the model showing TP (true positive) / TN (true negative) correct classifications and FP (false positive) / FN (false negative) false classifications are analyzed. Accuracy measures the model’s ability to accurately classify samples. In addition, sensitivity is the percentage of correctly classified actual positives, while specificity shows how well negative examples are predicted by the model.

(1) $$Accuracy\left(\%\right)=\left(\frac{TP+TN}{TP+TN+FP+FN}\right)*100$$

(2) $$Sensitivity\left(\%\right)=\left(\frac{TP}{TP+FN}\right)*100$$

(3) $$Specificity\left(\%\right)=\left(\frac{TN}{TN+FP}\right)*100$$

## 3. Results and Discussion

In literature, many recent works focus on studies of thyroid images [1,10,11,12,13,14,15,26,27,28,29]. In those studies, it was mentioned that the image processing skills of artificial intelligence methods and experienced/inexperienced physicians are compared, or the image processing skills of newly proposed artificial intelligence methods are compared with other artificial intelligence methods.

Unlike the available studies in the literature, in this study, sonographic images of thyroid nodules instead of image processing have been retrospectively evaluated, and the sonographic signs of the nodules have been determined as original by a specialist sonographer with more than 25 years of experience in thyroid sonography. Rather than using the limited number of US sonographic signs in the guides used in the literature, sonographic signs have been viewed on a very wide scale in the present study. The relationships between these sonographic signs and their sub-features have been investigated. Based on these relations, a new perspective has been proposed by using an artificial intelligence-based method, which is very different from the methods of creating guides in comparison to the previous studies.

Classification results have been obtained for the histopathological differential diagnosis of malignant/benign nodules within the scope of 27 sonographic signs determined as original. Classification results from ANFIS models trained with HB (ANFIS-HB), BP (ANFIS-BP), and GA (ANFIS-GA), as well as the DNN, are presented in Table 2. As depicted by the AC, SN, and SP values obtained from these methods, it has been found that the proposed ANFIS-GA method is more successful compared to both the derivative-based algorithms and the DNN method in the classification of malignant/benign nodules.

Advanced analyses of 27 signs can be simplified by revealing which features are most important. Methods such as Decision Trees (DT), Random Forests (RF), Linear Regression (LR), and Chi-Square (CS) can quickly reveal which features are of great importance at this point. Among these classification methods, the DT method is a commonly used classification method in data mining. The decision tree is a flow chart in the form of a tree structure. Each inner node corresponds to a decision on a sign, each branch indicates a result of the decision, and the leaf nodes represent classes in this flowchart. It uses the Gini Index or Knowledge Acquisition to determine which features are the most important ones, and then the most important signs are placed at the top of the decision tree. This process is repeated until all of the significant signs have been determined.

**Figure 4:**
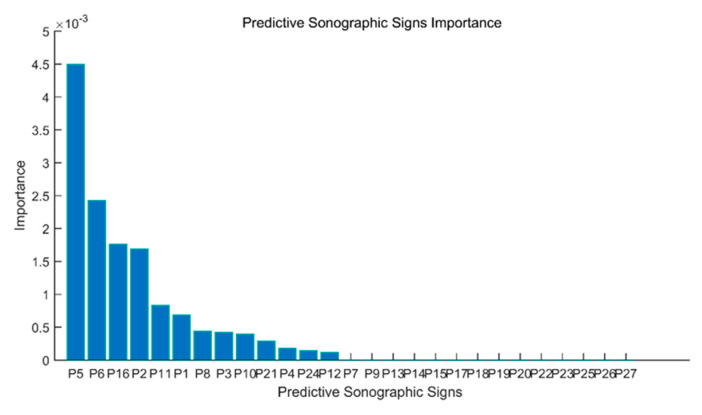
Rank values of 27 sonographic signs.

The details of the application of the DT algorithm to increase the performance of the proposed method are as follows. The decision tree algorithm is applied to all 27 sonographic signs (P1, P2, P3, …, P27), and it is found that the use of 13 sonographic signs given in the order of importance in Figure 4 in the differential diagnosis of thyroid nodules increases the efficiency the most. In other words, the rank value of each of the 27 sonographic signs with the DT algorithm is provided in Figure 4.

Simultaneously, the sonographer is asked to simplify the 27 sonographic signs by identifying the most significant ones based on his experience and only 8 sonographic signs are determined. Of these 8 US signs determined by the sonographer independently of the decision tree algorithm, 6 signs other than “doppler pattern”, “capsule relationship”, “size”, “number and distribution of internal microechogenicity, if any”, “AP/horizontal diameter ratio for taller-than-wide nodules”, “localization within thyroid lobe” and “free microechogenicity in the parenchyma” are found to be overlapping with 13 sonographic signs obtained by the decision tree algorithm. These results show the compatibility of the decision tree algorithm with the expert sonographer physician, who was accepted as the gold standard.

In addition, classification studies are carried out for the histopathological differential diagnosis of malignant/benign nodules within the scope of 13 significant sonographic signs determined with the use of the DT from 27 sonographic signs and 8 sonographic signs selected by the sonographer with the expert opinion within the scope of this study. The results obtained from both approaches are given in Table 3 and Table 4. For both approaches, it is observed that the proposed ANFIS-GA method is more successful in comparison to the derivative-based algorithms and the DNN method.

The average overall performance of the ANFIS-GA method, which is proposed as an innovative approach, on all dataset samples prepared with different data splitting methods and containing a different number of signs of 27, 13, and 8, is also detailed in Table 5 within the scope of the study. Thus, the overall performance of the proposed method has been examined against the DNN method that has been very popular in recent years.

The results provided in Table 5 imply that the average overall performance of the proposed method for all measurements is more successful compared to the derivative-based algorithms and the DNN method. It is found that the ANFIS-GA performs the best classification on the dataset containing all 27 signs and on the new datasets, including 13 and 8 signs obtained as a result of feature reduction with different methods with a mean classification AC value of 87.18%. In addition, the fact that the average general SN value of 78.76% and SP value of 90.66% performances of the proposed method yields better performance compared to the other methods also implies that the proposed method is fairly robust and reliable to be used with the purpose of such complex classification work.

**Figure 5:**
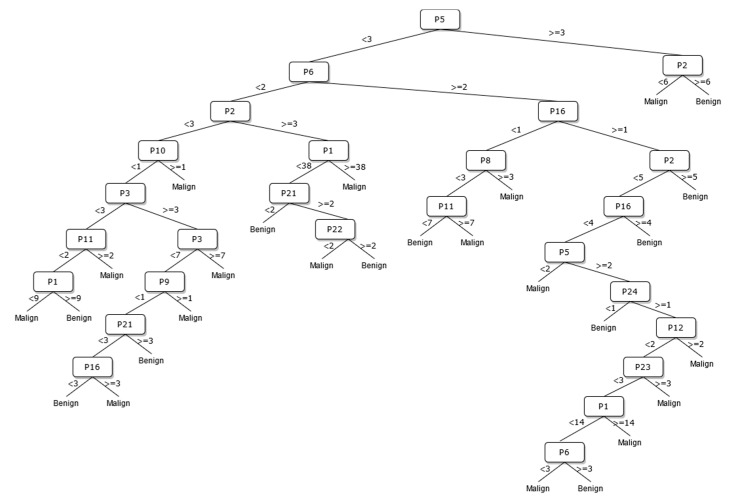
The DT of 27 sonographic signs.

**Figure 6:**
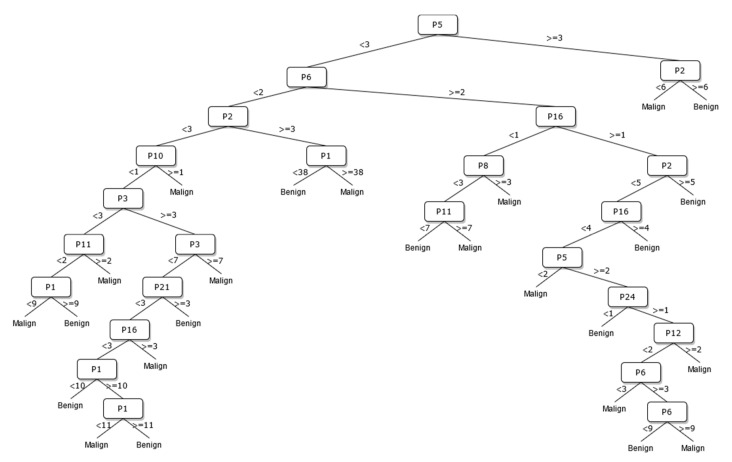
The DT of 13 sonographic signs.

**Figure 7:**
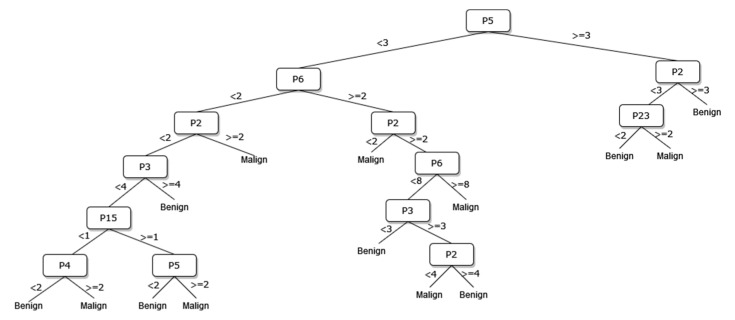
The DT of 8 sonographic signs.

A new DT model, which is not available in the current risk stratification models in the literature, is proposed as a guideline to help sonographers in the differential diagnosis of malignant/benign nodules. The DT flow diagrams have been created with 3 different datasets of 27, 13, and 8 sonographic signs that were determined and used by certain analyses for classification processes in the first part of the simulation studies. In addition, the classification performances of these diagrams are provided in Figure 5, Figure 6 and Figure 7. In this way, a guideline has been created to determine the malignancy of the nodule to the physician who examined the nodule with these DTs formed according to the sub-features of the sonographic signs of the nodules. Therefore, the recommended guideline is obtained with artificial intelligence methods contrary to the expert opinions in the literature and the clinical experience of expert sonographers.

DT works, questioning one variable at a time, to find the best split between various classes in the data. The threshold governs the choice to turn a projected probability or score into a class label. The measure of impurity (density of points of each class) of the data before splitting vs. the average measure of impurity of the splits were checked to choose the thresholds that give the best splits of the DT model used in this study. Mathematically, this case is defined as the “Gini Impurity”. This process is repeated on each of the split data until all splits contain pure samples (have an impurity of zero). In the end, all selected thresholds were combined to form a chain (tree) of rules for classifying the data.

**Figure 8:**
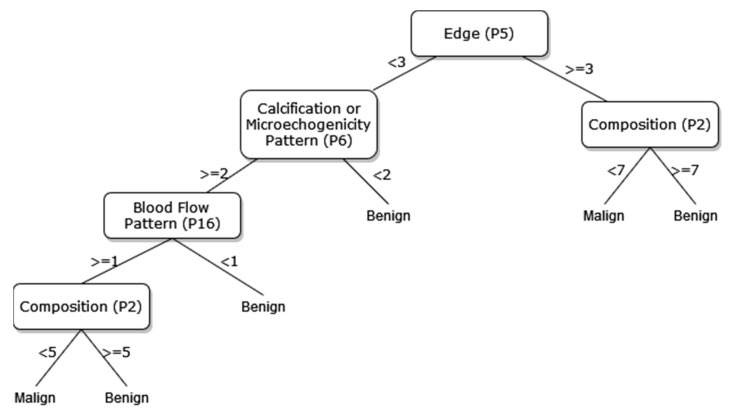
The pruned DT of 27 sonographic signs.

**Figure 9:**
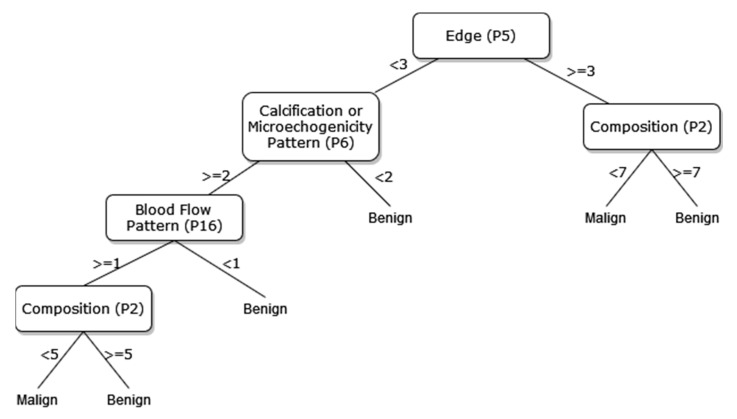
The pruned DT of 13 sonographic signs.

**Figure 10:**
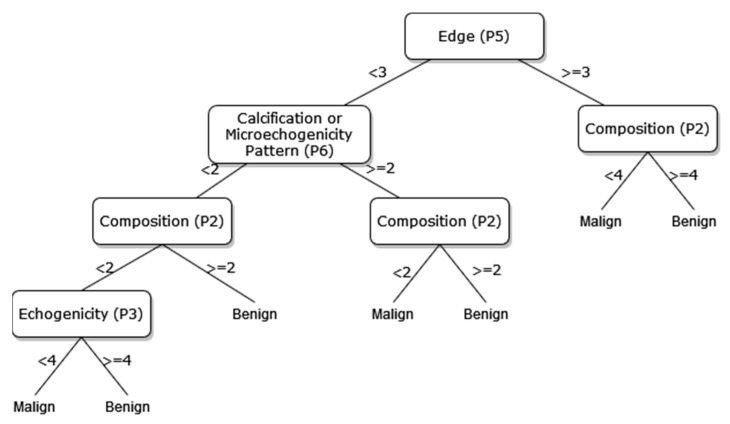
The pruned decision tree of 8 sonographic signs.

The process of removing the predictive signs in the branches that do not contribute significantly to the accurate classification rate of the DT is called pruning. The DT becomes both simpler and more understandable after the removal of such signs. The pruning process can be accomplished in two stages. In the first stage, called prepruning, step-by-step branching can be utilized by examining which sign is better in terms of the prediction power of the model by considering the predictive signs one by one without making any distinction. The second stage is called postpruning, in which the branches of a completed DT that do not contribute to the model are identified and removed from the model. The prepruning process, of which the maximum number of splits is 7, is applied to the DTs shown in Figure 8, Figure 9 and Figure 10 in order to come up with a simpler guideline to help the physician performing the nodule examination for the malignancy of the nodule. The obtained pruned DT diagrams are provided in Figure 8, Figure 9 and Figure 10.

The way the guide works can be summarized as follows: P1, P2, P3, etc. values in Figure 5, Figure 6, Figure 7, Figure 8, Figure 9 and Figure 10 refer to the abbreviations (P1: Size, P2: Composition, P3: Echogenicity, etc.) of sonographic signs in Table 1. Additionally, the numbers on the branches correspond to the Order No given in Table 1 for the sub-features of each sonographic sign.

In addition, the classification performances of the simpler and more understandable new DT diagrams obtained after the pruning process are compared within the scope of this study. The classification loss error values of all diagrams and pruned diagrams obtained by the DT are tabulated in Table 6 in this context.

As depicted from the results provided in Table 6, the DT flow diagram model given in Figure 6 created with 13 sonographic signs yields the best performance. However, DT diagram model number 6, which is created with 8 sonographic signs by applying the pruning process, is much less complex, and the classification loss error value is slightly less than the best one (see Table 6). DT diagram model number 6 is much more understandable and much less complex with acceptable performance; all of these suggest that it can be considered a good guideline for physicians who examine the nodule to determine the malignancy of the nodule in this respect. The application DT used in this study is explained in Figure 10, which is the diagram of model number 6. In short, the values of the sonographic signs sub-features (see Table 1) are checked against the branch values of DT given in Figure 10, and this process continues from the root node to the leaf node. Finally, the malignant/benign differential diagnosis of the nodule is determined according to the reached leaf node’s branch value.

## 4. Conclusions

Detailed simulation studies have been performed by using the sonographic signs of the thyroid nodules of patients whose diagnoses are confirmed histopathologically in this study. An innovative approach, such as training the ANFIS model with the GA algorithm, has been applied in the differential diagnosis of malignant/benign nodules considered as a real-world problem. In addition, the DT has been utilized to determine the most effective and significant signs in the differential diagnosis of malignant/benign nodules. Thus, a newly proposed guideline introduced in this paper, for which further validation should be confirmed in a larger study group, shows the effect of the sonographic sign on the differential diagnosis of malignant/benign nodules.

The current guidelines in the literature generally offer the usage of grayscale US signs for the differentiation of benign nodules from malignant ones. The three US signs that can be considered the most important are a taller-than-wide pattern, microcalcifications, and border irregularity, especially when they exist in solid and hypoechoic nodules. However, there might be some other possibly important US signs, such as the absence of a halo, a thick and irregular halo, and blood flow pattern. More importantly, as our DT guide suggests, the relations between all US signs could be more complex than we thought and could possibly be more helpful for better differential diagnosis. Testing our proposed method in a larger database is needed to discover the real potential of machine learning and deep learning methods that take all possible US signs and relations between them into account in classifications of thyroid nodules.

It has once again been observed that the GA algorithm is more successful compared to the derivative-based algorithms in the training of ANFIS parameters. Optimizing the ANFIS structure, which is a difficult problem with a population-based algorithm, shows the importance of metaheuristic algorithms. In addition, the performance of the proposed ANFIS-GA method has been compared to the performances of derivative-based and the DNN methods, which are very popular in recent years and have been applied in many areas, and better results have been obtained. This study has revealed that the proposed method is robust and reliable with the results obtained from different measurements used in the comparison process. Thus, we plan to apply the proposed method to real-world problems in different areas in the future.

## Figures and Tables

**Table 1:** 27 Sonographic signs of nodules evaluated in study group.

Order	Notation	Sonographic Sign	Value	Sub-Features
1	P1	Size (mm)	-	-
2	P2	Composition	1	Completely solid
			2	Solid-containing microcysts (<5%)
			3	Predominantly solid (5–10% cystic)
			4	Predominantly solid (10–25% cystic)
			5	Predominantly solid (25–50% cystic)
			6	Predominantly cystic (50–90% cystic)
			7	Almost completely cystic (>90%)
			8	Completely cystic
			9	Spongiform
3	P3	Echogenicity	1	Anechoic
			2	Markedly hypoechoic
			3	Hypoechoic
			4	Isoechoic
			5	Hyperechoic
			6	Mixed dominant hyperechoic
			7	Mixed dominant hypoechoic
			8	Mixed dominant isoechoic
			9	Unable to be evaluated (calcified nodule)
4	P4	Shape (AnteroPosterior/Transvers diameter)	1	No
			2	Yes
5	P5	Edge	1	Ill-defined
			2	Smooth
			3	Irregular (coarse lobulation)
			4	Irregular (microlobulation)
			5	Irregular (spiculated)
			6	Ill-defined and irregular
			7	Mass appearance without borders
			8	Extrathyroidal extension
6	P6	Calcification or Microechogenicity Pattern	1	No
			2	Punctate
			3	Linear
			4	Punctate and linear
			5	Having cluster calcifications and acoustic shading
			6	V-shaped (comet tail)
			7	Internal macrocalcification
			8	Incomplete peripheral calcification
			9	Complete peripheral calcification
			10	Incomplete peripheral calcification and internal linear and punctate calcification
			11	Internal coarse echogenicities without punctate, linear, and acoustic shading
			12	Coarse internal echogenicities (no acoustic shading)
			13	Punctate, linear, and coarse calcification
			14	Coarse internal calcification and coarse echogenicities
7	P7	Distribution of Linear Microechogenicity, If Any	1	In the posterior of the cystic areas
			2	In solid areas
			3	In mixed pattern
8	P8	Number and Distribution of Internal Microechogenicity, If Any	1	Rare (<5)
			2	Multiple scattered (≥5)
			3	Multiple peripheral (≥5)
			4	Snowstorm
9	P9	Inner Echo If There Is Incomplete Peripheral Calcification	1	Hyperechoic
			2	Isoechoic
			3	Hypoechoic
10	P10	AP/Horizontal Diameter Ratio for Taller-Than-Wide Nodules	-	-
11	P11	Capsule Relationship	1	No
			2	In the anterior
			3	In the posterior
			4	In the upper pole
			5	In the lower pole
			6	Sitting in the upper pole
			7	Sitting in the lower pole
			8	Extended areas
			9	Lateral
12	P12	The Percentage of Perimeter Contacting the Capsule If There Is a Capsule Relationship	1	10%
			2	20%
			3	30%
			4	40% and above
13	P13	Abutement of thyroid Capsule If There Is a Capsule Relationship	1	No
			2	Mild
			3	Marked
14	P14	Interruption in Echogenic Capsule If There is a Capsule Relationship	1	No
			2	Yes
			3	Gross extrathyroidal
			4	Undefined
15	P15	Halo	1	No
			2	Yes (indistinct)
			3	Yes (fine smooth)
			4	Yes (thick or asymmetrical)
16	P16	Blood Flow Pattern	1	No blood flow
			2	Predominantly peripheral
			3	Internal or mixed
			4	Inferno (intense internal)
17	P17	Reflective Shadowing in Solid Nodule	1	Yes
			2	No
18	P18	Posterior Acoustic Enhancement in Solid Nodule	1	Yes
			2	No
19	P19	Solid Component Features If Malignant Nodule Is Cystic	1	Eccentric solid component
			2	Microechogenicity in the solid component
			3	No feature
			4	Eccentric solid component and microechogenicity
20	P20	Localization	1	Right
			2	Left
			3	Isthmus
			4	Pyramidal lobe
21	P21	Localization within thyroid lobe	1	Upper (1/3)
			2	Intermediate (1/3)
			3	Lower (1/3)
			4	Largely filling the lobe
22	P22	Solitary/Multinodular	1	Single
			2	Multiple
23	P23	US signs suggesting Chronic Thyroiditis	1	No
			2	Suspicious
			3	Typical
24	P24	Free Microechogenicity in the Parenchyma	1	No
			2	Yes
25	P25	Size and Features of Central Lymph Node in USG	1	No
			2	Nonspecific (<5 mm)
			3	Nonspecific (5–10 mm)
			4	Nonspecific (>10 mm)
			5	Suspicious (<5 mm)
			6	Suspicious (5–10 mm)
			7	Suspicious (>10 mm)
			8	Typical metastatic (<5 mm)
			9	Typical metastatic (5–10 mm)
			10	Typical metastatic (>10 mm)
26	P26	Number of Central Lymph Nodes in USG	1	Single
			2	Several (2–4)
			3	Multiple (>5)
27	P27	Lateral Metastatic Lymph Node in USG	1	No
			2	Suspicious
			3	Typical

**Table 2:** The performance of the proposed method compared to conventional methods for 27 signs.

Classifier		ANFIS-HB		ANFIS-BP		DNN		ANFIS-GA	
Data Splitting Methods	%70–30 splitting	AC	74.79	AC	84.87	AC	84.99	AC	89.08
		SN	65.63	SN	71.79	SN	76.65	SN	89.29
		SP	78.16	SP	91.25	SP	89.23	SP	89.01
	%80–20 splitting	AC	81.25	AC	85.00	AC	87.50	AC	88.75
		SN	72.22	SN	66.67	SN	77.84	SN	83.33
		SP	83.87	SP	90.32	SP	92.44	SP	90.32
	10-fold validation	AC	81.38	AC	82.68	AC	87.65	AC	85.68
		SN	69.82	SN	64.74	SN	78.08	SN	74.99
		SP	86.06	SP	90.60	SP	91.76	SP	89.93
	5-fold validation	AC	78.86	AC	82.92	AC	88.19	AC	86.42
		SN	65.80	SN	65.83	SN	75.88	SN	79.96
		SP	84.28	SP	89.76	SP	93.03	SP	89.58

**Table 3:** The performance of the proposed method compared to conventional methods for 13 signs.

Classifier		ANFIS-HB		ANFIS-BP		DNN		ANFIS-GA	
Data Splitting Methods	%70–30 splitting	AC	80.67	AC	84.87	AC	85.83	AC	89.92
		SN	66.67	SN	72.73	SN	74.60	SN	84.85
		SP	86.05	SP	89.53	SP	91.62	SP	91.86
	%80–20 splitting	AC	82.50	AC	86.25	AC	88.75	AC	90.00
		SN	76.00	SN	80.00	SN	80.92	SN	84.00
		SP	85.45	SP	89.09	SP	92.72	SP	92.73
	10-fold validation	AC	82.64	AC	88.49	AC	88.65	AC	88.67
		SN	74.17	SN	82.04	SN	80.32	SN	79.77
		SP	86.26	SP	91.33	SP	91.34	SP	92.59
	5-fold validation	AC	80.15	AC	84.17	AC	88.71	AC	88.42
		SN	66.95	SN	67.70	SN	78.36	SN	75.03
		SP	85.58	SP	90.86	SP	92.77	SP	93.61

**Table 4:** The performance of the proposed method compared to conventional methods for 8 signs.

Classifier		ANFIS-HB		ANFIS-BP		DNN		ANFIS-GA	
Data Splitting Methods	%70–30 splitting	AC	73.11	AC	76.47	AC	82.49	AC	84.87
		SN	47.37	SN	55.26	SN	79.70	SN	73.68
		SP	85.19	SP	86.42	SP	83.84	SP	90.12
	%80–20 splitting	AC	78.75	AC	85.00	AC	83.75	AC	86.25
		SN	55.56	SN	61.11	SN	81.84	SN	83.33
		SP	85.48	SP	91.94	SP	84.59	SP	87.10
	10-fold validation	AC	77.88	AC	81.89	AC	84.60	AC	83.66
		SN	58.70	SN	63.71	SN	73.52	SN	71.20
		SP	85.65	SP	88.61	SP	89.47	SP	89.16
	5-fold validation	AC	79.63	AC	83.43	AC	85.16	AC	84.44
		SN	59.75	SN	64.78	SN	74.47	SN	65.65
		SP	87.28	SP	90.90	SP	90.23	SP	91.92

**Table 5:** The average performance criteria for all methods.

	Measurements (%)		
Classifier	AC	SN	SP
ANFIS-HB	79.30	64.89	84.94
ANFIS-BP	83.84	68.03	90.05
DNN	86.36	77.68	90.25
ANFIS-GA	87.18	78.76	90.66

**Table 6:** The comparison of classification loss error values obtained from different DT diagrams.

Model No	Models	Pruning	Number of Splits	Classification Loss Error
1	DT with 27 predictor	No	27	0.1457
2	DT with 13 predictor	No	13	0.1332
3	DT with 8 predictor	No	8	0.1658
4	DT with 27 predictor	Yes	7	0.2286
5	DT with 13 predictor	Yes	7	0.2161
6	DT with 8 predictor	Yes	7	0.1910

## Data Availability

The code will be made available on request.
